# Biochemical evidence for relaxed substrate specificity of Nα-acetyltransferase (*Rv3420c*/*rimI*) of *Mycobacterium tuberculosis*

**DOI:** 10.1038/srep28892

**Published:** 2016-06-29

**Authors:** Deepika Pathak, Aadil Hussain Bhat, Vandana Sapehia, Jagdish Rai, Alka Rao

**Affiliations:** 1 CSIR-Institute of Microbial Technology, Sector 39-A, Chandigarh, 160036, India; 2 Institute of Forensic Science & Criminology, Panjab University, Sector 14, Chandigarh, 160014, India

## Abstract

Nα-acetylation is a naturally occurring irreversible modification of N-termini of proteins catalyzed by Nα-acetyltransferases (NATs). Although present in all three domains of life, it is little understood in bacteria. The functional grouping of NATs into six types NatA - NatF, in eukaryotes is based on subunit requirements and stringent substrate specificities. Bacterial orthologs are phylogenetically divergent from eukaryotic NATs, and only a couple of them are characterized biochemically. Accordingly, not much is known about their substrate specificities. *Rv3420c* of *Mycobacterium tuberculosis* is a NAT ortholog coding for RimI^Mtb^. Using *in vitro* peptide-based enzyme assays and mass-spectrometry methods, we provide evidence that RimI^Mtb^ is a protein Nα-acetyltransferase of relaxed substrate specificity mimicking substrate specificities of eukaryotic NatA, NatC and most competently that of NatE. Also, hitherto unknown acetylation of residues namely, Asp, Glu, Tyr and Leu by a bacterial NAT (RimI^Mtb^) is elucidated, *in vitro*. Based on *in vivo* acetylation status, *in vitro* assay results and genetic context, a plausible cellular substrate for RimI^Mtb^ is proposed.

Appreciated once in eukaryotes only, Nα-acetylation of proteins appears quintessential in all three domains of life. It is implicated in several cellular functions in eukaryotes including its role as a multifunctional regulator, protein degradation signal, regulator of protein translocation to ER, in protein-protein interactions as well as genetic defects and cancer in human^1^,^2^,^3^. Nα-acetylated proteins range from 50–90% in eukaryotes^1^ and 10–28% in archaea and bacteria^4^,^5^,^6^,^7^,^8^.

N-terminal acetylation (Nα-acetylation) is part of N-terminal protein processing events in a cellular context. The enzymes instrumental in acetylation of a diverse range of cellular proteins are known as Nα-acetyltransferases (NATs). Using an activated acetyl coA donor, NATs catalyze an irreversible transfer of the acetyl moiety on to the free N-terminus of a nascent growing polypeptide, usually in a co-translational manner^9^. Using protein sequences of catalytic units of NATs known in yeast, Polevoda and Sherman identified orthologs in model eukaryotic and prokaryotic genomes and then classified these (phylogenetic) into six protein families namely: Ard1p; Nat3p; Mak3p; CAM; BAA; and Nat5p. According to this, bacterial and archaeal NATs were found divergent from eukaryotic NATs and classified under BAA family^10^. Subsequently, based on subunit requirements and substrate specificities (N-terminal amino acid sequence of acceptor polypeptide), at least, six different NATs/NAT complexes are described, namely, NatA (N- Ser/Ala/Thr/Gly/Val/Cys), NatB (N-Met-Asp/Glu/Asn), NatC (N-Met-Ile, Leu, or Phe), NatD (Histones), NatE and NatF (NatF is not known in prokaryotes and found in metazoans only). NatE and NatF specificities overlap that of NatC and differ primarily in the requirement of subunits^9^.

While the presence of NATs in *Escherichia coli* (*E. coli*) is acknowledged since 1987 11,12, only seven proteins were identified as Nα-acetylated proteins in prokaryotes till 2007 13. Accordingly NATs are little understood in bacteria. RimL, RimJ and RimI are the best-known examples of bacterial NATs catalyzing Nα-acetylation of ribosomal proteins L7/L12 (at Ser-Ile-), S5 (at Ala-His-) and S18 (at Ala-Arg-), respectively. Therefore, these enzymes are identified and named strictly as per their substrate specificities^14^. Only a couple, namely, RimL^15^ and RimI^13^ are characterized biochemically in *Salmonella typhimurium* (*S. typhimurium*). Despite good conservation across bacterial genomes, sequence similarity among RimI, RimJ and RimL is very poor. In bacteria, a posttranslational mode of Nα-acetylation is indicated. However, unequivocal *in vitro* proof for a posttranslational Nα-acetylation has been established for RimL^15^ alone, that acetylates N-terminal Ser residue of purified full-length ribosomal protein L12.

*Mycobacterium tuberculosis* (*Mtb*) is the etiological agent of Tuberculosis. Posttranslational modifications of proteins are implicated in the virulence of this pathogen^16^. Lysine side chain acetylation of proteins is known for quite some time in *Mtb*, but acetylation of N-termini of proteins is taken note of, only recently. The first reported Nα-acetylated protein of *Mtb*, EsxA (ESAT-6, for early secreted antigenic target, 6 kDa), is part of *Mtb* secretome in a human host cell. Its interaction with EsxB (CFP-10, for culture filtrate protein, 10 kDa) is an important determinant of virulence in *Mtb*^17^,^18^. The mechanism behind the Nα-acetylation of EsxA and how this modification affects Esx-1-mediated secretion and virulence are unknown. In a more recent study, using transposon insertion mutagenesis of a non-cytotoxic *Mycobacterium marinum* strain a putative NAT (*MMAR_0039* gene) is identified as protein responsible for homeostasis of EsxA Nα-acetylation. It also proposes an inverse correlation between EsxA acetylation and virulence^19^. However, our sequence searches did not reveal any corresponding homolog of MMAR_0039 in *Mtb*.

In two isolated studies that were aimed at annotating translational start sites (TSS), as many as 253 out of a total 874 peptides were identified to be Nα-acetylated in *Mtb*^6^,^20^. This indicates that at least 28% proteins constitute *Mtb* Nα-acetylome. Further, many of these proteins are important for virulence and form *Mtb* secretome in human macrophage cell^21^. Sequence analysis of mycobacterial Nα-acetylated peptides suggests the presence of at least one NAT with eukaryotic NatA-like substrate specificity that is involved in acetylating approximately 84% of the protein substrates (Supplementary Figure S1). The closest homolog of NatA catalytic subunit Ard1 is RimI, in bacteria^10^, sharing 31% similarity. As described already, bacterial Rim enzymes are considered ribosomal protein (substrate) specific. Therefore, it was intriguing to see if protein RimI^Mtb^ could acetylate ribosomal proteins or additional substrates in *Mtb*.

Interestingly, gene *Rv3420c* encoding RimI^Mtb^ is present in a genomic context that is conserved in 20% of bacterial genomes^22^. Most of the genes clustered along with *rimI*, namely *groEL1* and *groES* (chaperone encoding genes) and *tsaD*, *tsaB,* and *tsaE* genes (encoding homologs of the tRNA-A_37_-t^6^A transferase enzymes)^23^ are essential for *Mtb* survival and/or pathogenesis as indicated in Tuberculist database^24^. Therefore, we were interested in investigating the functional role of RimI^Mtb^.

## Results

### RimI^Mtb^ is a monomer in solution

**Figure 1:**
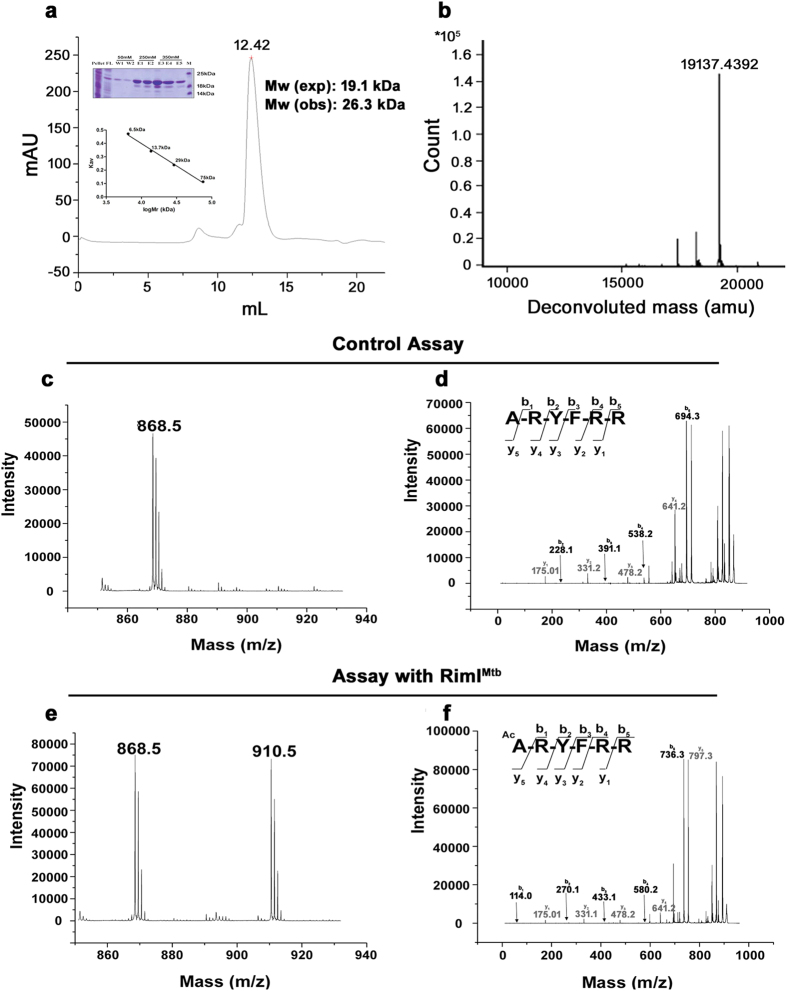
Purification of RimI^Mtb^ and identification of Nα-acetyltransferase activity of RimI^Mtb^. (**a**) Gel filtration of RimI^Mtb^ using superdex 75 10/300GL. Ni-NTA purified RimI^Mtb^ (as shown in protein gel) eluted as monomer (calibration curve given in inset) (**b**) Confirmation of intact mass (19.1 kDa) of purified RimI^Mtb^ monomer using LC-ESI-MS (**c**) MS analysis of control assay (without enzyme) using DPC peptide (ARYFRR) as substrate (**d**) MS/MS analysis of precursor ion (868.5 Da) of DPC peptide in control assay (**e**) MS analysis of enzyme assay confirming acetylation of DPC peptide by RimI^Mtb^. Modified peptide was observed (910.5) with an increase of 42.0105 Da as compared to unmodified peptide (868.5 Da) concomitant to the addition of acetyl group (**f**) MS/MS analysis of modified (910.5 Da) precursor ion of the substrate (DPC) peptide. An increase of 42.0105 Da was observed in all the b-ions (in black) while the y-ions (in grey) remained the same as that of unmodified substrate, confirming the site of acetylation as the N-terminal amino acid i.e. Ala.

*Rv3420c* from *Mtb* was cloned, expressed and purified as a C-terminal His-tagged protein (RimI^Mtb^) (Materials and Methods, Supplementary Tables S1 and S2). Gel filtration chromatography and intact mass analysis of purified protein by LC-ESI-MS suggested that RimI^Mtb^ is a monomer of 19.1 kDa (Fig. 1a,b). The identity of the protein was confirmed by peptide mass fingerprinting.

### RimI^Mtb^ acetylates N -terminus of rpS18 (ribosomal protein)

Ribosomal protein S18 (rpS18) is the only known substrate of RimI in bacteria namely, *E. coli*^12^ and *S. typhimurium*^13^. *Mtb* genome contains two putative ORFs for ribosomal protein S18 namely, *Rv0055* (rpsR1) and *Rv2055* (rpsR2). Therefore, acetyltransferase activity of RimI^Mtb^ was identified and assessed using substrate peptides representing first six residues (except N terminal-Met) of rpS18 as well as rpsR1 and rpsR2 (Table 1). A clear mass increase of 42.0105 Da was identified in rpS18 that was concomitant with the addition of an acetyl group (Fig. 1c,e). Further, MS/MS analysis confirmed the site of acetylation as the N-terminal amino acid (Fig. 1d,f). However, N-terminus of rpsR1 (DP1) and rpsR2 (DP2) were not acetylated efficiently (Supplementary Figure S2). Therefore, acetylation of *Mtb* ribosomal proteins at N-terminal Ala could not be ascertained. Our result reinforces previous observations that Nα-acetylation signatures are not absolute and downstream residue differences might bring variable outcomes^9^.

### Relaxed acceptor substrate specificity of RimI^Mtb^

**Figure 2:**
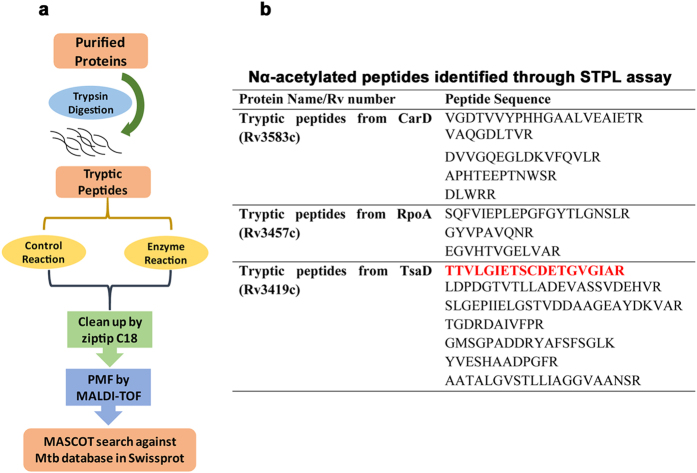
Small-Scale Tryptic Peptide Library (STPL) assay. (**a**) Schematic representation of STPL assay (**b**) Sequences of peptides (belonging to tryptic digests of CarD, RpoA and TsaD) Nα-acetylated at neo-terminal amino acid residues by RimI^Mtb^, *in vitro*. Nα-acetylated N-terminus of TsaD is highlighted in red.

Protein sequence analysis of the Nα-acetylome of *Mtb*^6^ reveals that at least 54% of proteins have Thr at their N-terminus followed by Ser in 19% and Met in 12% of the proteins. In other words, *Mtb* Nα-acetylome primarily represents substrates preferred by eukaryotic NatA (Thr, Ser and Ala termini) and NatE (Met termini) (Supplementary Figure S1). Therefore, to explore the substrate specificity of RimI^Mtb^, a small-scale tryptic peptide library (STPL) assay was developed (Fig. 2a, described in detail in Materials and Methods). Several peptides were detected as acetylated at their neo N-termini by RimI^Mtb^ while the same remained un-acetylated in corresponding control (without RimI^Mtb^) assays (Fig. 2b). N-terminal residues identified acetylated include Ala, Ser, Val, Leu, Tyr, Asp and Glu. While Ala and Ser are well known NatA substrates, Leu and Tyr are hitherto unidentified. Asp and Glu residues are modified by Naa10p catalytic subunit alone when not in complex with its auxiliary unit Naa15p^9^. From the results of STPL assays, we conclude that RimI^Mtb^ has relaxed specificity *in vitro* and it is promiscuous to substrates varying in length as well as sequence (Fig. 2b).

**Figure 3:**
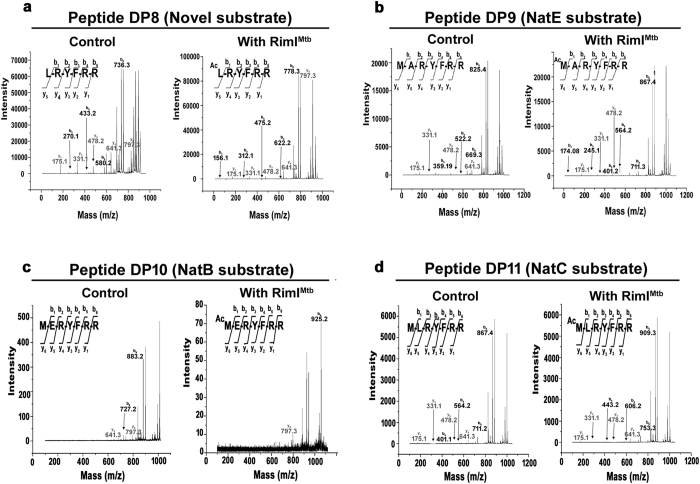
Relaxed substrate specificity of RimI^Mtb^. Results of NAT assays with substrate peptides DP8, DP9, DP10 and DP11 analyzed using MALDI-TOF/TOF. (**a**) MS/MS of 910.5 Da precursor ion (S/N = 4201) of unmodified and 952.5 Da precursor ion (S/N = 5894) of modified substrate peptide representing novel substrate specificity of RimI^Mtb^ (**b**) MS/MS of 999.5 Da precursor ion (S/N = 7037) of unmodified and 1041.5 Da precursor ion (S/N = 4256) of modified substrate peptide representing N-terminus of conventional NatE substrate (**c**) MS/MS of 1057.8 Da precursor ion (S/N = 15916) of unmodified and 1099.5 Da precursor ion (S/N = 1011) of modified substrate peptide representing N-terminus of conventional NatB substrate (**d**) MS/MS of 1041.5 Da precursor ion (S/N=17767) of unmodified and 1083.5 Da precursor ion (S/N = 14629) of modified substrate peptide representing N-terminus of conventional NatC substrate.

**Figure 4:**
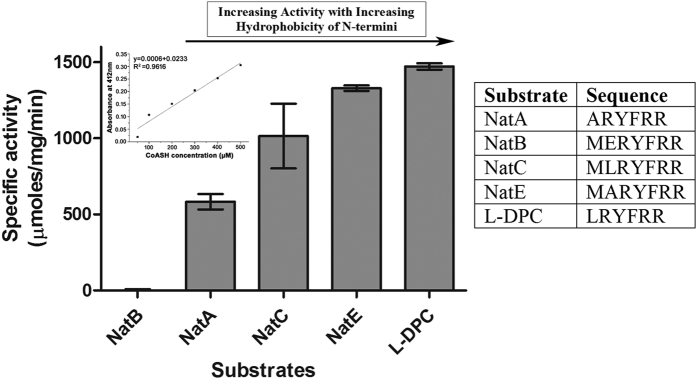
Determination of specific activity of RimI^Mtb^ towards peptide substrates using DTNB assay. The amount of product (CoASH) generated as a result of acetyl transfer was monitored at 412 nm. The concentration of the product was quantified using CoASH calibration curve (inset) and specific activity of RimI^Mtb^ against each substrate plotted in terms of μmoles/mg/min. The results shown here represent mean ± SD of experiments performed in triplicate.

To decipher the precise substrate preference of RimI^Mtb^, substrate peptide DPC (NatA substrate) was custom synthesized with single residue modifications at its N-terminus to represent substrate specificities of NatE (DP9), NatB (DP10), NatC (DP11), and novel substrate Leu (DP8) and tested, accordingly (Table 1). All the peptides were modified by RimI^Mtb^ (Fig. 3). A relative comparison of the ratios of intensities of modified *vs.* unmodified substrate peptides (having similar ionization efficiencies and reactions carried out under identical conditions) revealed that RimI^Mtb^, in fact, could acetylate DP9 (NatE substrate) 18 fold better than DPC (NatA substrate) (Supplementary Table S3). Further, to ascertain the specificity of the enzyme, quantitative DTNB assays were conducted (as described in Materials and Methods) and specific activity of RimI^Mtb^ against typical NAT substrates was determined. As observed in Fig. 4, DTNB assay clearly confirmed the relaxed specificity of RimI^Mtb^, encompassing that of at least three major eukaryotic NATs namely, NatA, NatC and NatE. However, RimI^Mtb^ acetylated NatE substrate more efficiently than that of other NATs. Further, from DTNB assay results a positive correlation between hydrophobicity of N-terminal residue of the substrate and enzyme activity was observed (Fig. 4).

Kinetic assessment of RimI^Mtb^ catalyzed acetylation of peptide substrate was carried out by selected ion monitoring (SIM) of the acetylated product through LC-ESI-MS/MS, where DP8 (LRYFRR) acted as an acceptor (Supplementary Methods and Supplementary Figure S4). Acetylated-DPC (Ac-ARYFRR) was chosen as the internal standard for its similar molecular weight and ionization efficiency as that of the product. Obtained K_M_ (for DP8) 2.9 ± 0.9 mM indicated that acetylation efficiency of RimI^Mtb^ towards DP8 is similar to the efficiency observed previously in the case of RimI of *S. typhimurium* for Ala residue (2.2 ± 0.2 mM)^13^.

### Stringent donor substrate specificity of RimI^Mtb^

In addition to NAT activity, human and yeast NATs (Naa10p and Naa50p) are known to possess *in vitro* and *in vivo*, N-terminal-propionyltransferase activity^9^. Using acetyl coA or propionyl coA or succinyl coA as donor substrate while keeping the acceptor substrate identical (DP8 having sequence LRYFRR), RimI^Mtb^ was found to accept only acetyl coA as a donor (data not shown). Thus, RimI^Mtb^ has stringent donor substrate specificity in comparison to eukaryotic NATs.

### RimI^Mtb^ acetylates N-terminus of neighboring non-ribosomal proteins

**Figure 5:**
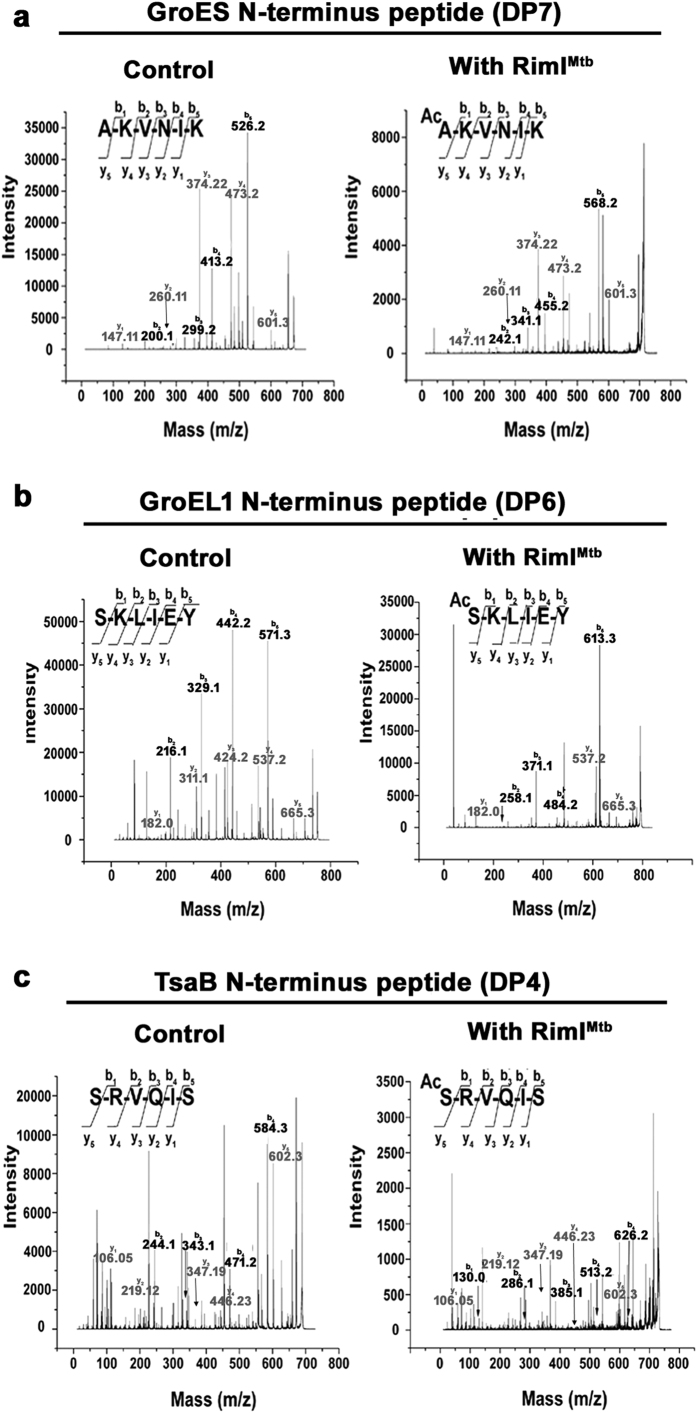
Acetylation of N-termini of neighboring proteins by RimI^Mtb^. (**a**) MS/MS of 672.4 Da precursor ion (S/N = 1999) of unmodified and 714.4 Da precursor ion (S/N = 557) of modified substrate peptide representing N-terminus of GroES (**b**) MS/MS of 752.3 Da precursor ion (S/N = 11634) of unmodified and 794.4 Da precursor ion (S/N = 1984) of modified substrate peptide representing N-terminus of GroEL1 (**c**) MS/MS of 689.4 Da precursor ion (S/N = 409) of unmodified and 731.3 Da precursor ion (S/N = 138.5) of modified substrate peptide representing N-terminus of TsaB.

RimI^Mtb^ is present in an interesting conserved genomic context composed of homologs of the tRNA-A_37_-t^6^A transferase enzymes and essential chaperone proteins GroES and GroEL1 (as discussed in the introduction). GroES is known to be Nα-acetylated in the mycobacterial proteome, previously^6^ while both GroES and GroEL are observed to be Nα-acetylated in *Pseudomonas aeruginosa*^8^. Using STPL assay (as described already), we had successfully identified the Nα-acetylation of TsaD N-terminus by RimI^Mtb^ (Fig. 2b highlighted in red). Keeping in view the relaxed specificity of RimI^Mtb^, we next explored if these neighboring proteins could be the substrate (s) for RimI^Mtb^. Therefore, peptides representing N-terminus of other neighboring proteins namely, TsaB (DP4), TsaE (DP5), GroEL1 (DP6) and GroES (DP7) were custom synthesized and tested as substrates. RimI^Mtb^ could acetylate peptides representing N-terminus of GroES and GroEL1 (Table 1 and Fig. 5 and Supplementary Figure S3). However, highest specific activity of RimI^Mtb^ (231.69 μmoles/mg/min) was observed against GroES peptide (DP7) alone (Supplementary Figure S3).

Having understood that RimI^Mtb^ could acetylate N-terminus of at least three of its neighboring proteins with varying efficiencies, GroES, and TsaB-TsaD (complex) were expressed and purified successfully from *E. coli*. Enzyme assays utilizing C_14_ labeled acetyl-coA (Perkin Elmer) were set up and analyzed by SDS-PAGE. Interestingly, none of these folded proteins could be acetylated by RimI^Mtb^. In the case of GroES, first 7–20 amino acid residues are known to be involved in multimerization^25^, therefore, might not be accessible to RimI^Mtb^ in folded conformation. However, nothing is known about the folded state of TsaB and TsaB-TsaD complex. This indicates that secondary structure of folded protein substrates could be an important determinant of Nα-acetylation activity of the enzyme as noted previously^10^.

### Plausible cellular substrate for RimI^Mtb^

In our experiments, we find that a) RimI^Mtb^ does acetylate peptides representing N-terminus of GroES, GroEL1, and TsaD proteins, *in vitro*; b) *rimI*, *groES*, *groEL1*, *tsaB, tsaE* and *tsaD* are co-regulated as part of a single operon (results are part of a separate study and communicated elsewhere); c) significant (P value < 0.05) specific activity of RimI^Mtb^ was observed against peptide representing N-terminus of GroES. Further, GroES and GroEL are identified Nα-acetylated in bacterial proteomes, previously^6^,^8^. Therefore, we propose GroES as one of the plausible cellular substrates for RimI^Mtb^ in *Mtb*. In eukaryotes, Nα-acetylation of GroES imparts increased resistance towards proteolytic degradation and longer half–life as compared to the non-acetylated (recombinant) counterpart^26^. However, nothing is yet understood about the role of such acetylation in bacteria.

### *In silico* modeling of RimI^Mtb^: a comparison viz a viz other known NATs

**Figure 6:**
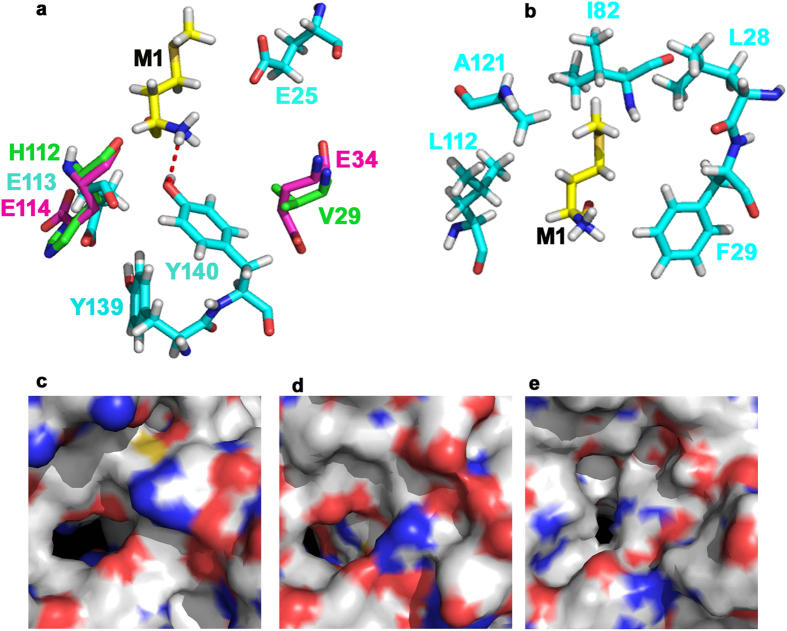
Structural alignment of RimI^Mtb^ model (docked with peptide DP9-MARYFRR) with crystal structures of Naa50p (NatE) and TvArd1. Cyan: RimI^Mtb^ model (developed using ITSSAR), Yellow: Substrate peptide DP9 (docked using Flexpepdock), Magenta: TvArd1 (4pV6) and Green: Naa50p (3TFY). (**a**) Key catalytic residues conserved between three structures and as listed in Supplementary Figure S5D, aligned at identical positions. Residues Y138 and Y139 (from Naa50p) and Y140 and Y141 (from TvArd1) (not shown in cartoon) and Y139 and Y140 of RimI^Mtb^ (shown in cartoon), aligned perfectly. E25 of RimI^Mtb^ is identified as distinctively placed in comparison with corresponding residues V29 of Naa50p and E34 of TvArd1. Hydrogen-bond between N-terminal Met of docked peptide and Y140 of RimI^Mtb^ is represented by dotted line (**b**) Residues involved in hydrophobic contact between RimI^Mtb^ and N-terminal Met of docked substrate peptide, defining substrate binding pocket of the enzyme (**c**) Surface cartoon of binding pocket of Naa50p/NatE (**d**) Surface cartoon of binding pocket of TvArd1 (**e**) Surface cartoon of binding pocket of RimI^Mtb^.

In bacteria, Rim enzymes possess stringent substrate specificities. They are known to acetylate Ala and Ser residues only that are conventional NatA substrates. It was interesting to observe that RimI^Mtb^ could acetylate a conventional NatE substrate (Met N-termini), more efficiently than the expected NatA substrates (Fig. 4). Therefore, we attempted *in silico* modeling of RimI^Mtb^ using ITSSAR^27^. The best model was selected based on lowest RMSD value that shared maximum similarity with TvArd1 of *Thermoplasma volcanium* (PDB ID: 4pV6)^28^. TvArd1 like its ortholog SsArd1 from *Sulfolobus solfataricus* (*S. solfataricus*) belongs to NatA family and acetylates Alba1 protein at N-terminal Ser residue. The selected model of RimI^Mtb^ was then docked with DP9 peptide (MARYFRR) (Supplementary Figure S5) using flexpepdock server^29^. Further, LPC/CSU server^30^ was employed to identify the key residues involved in stabilizing interactions, and contact analysis of substrate peptide docked on to the RimI^Mtb^ model. Sequence analysis and structural alignment of docked RimI^Mtb^, Naa50p (3TFY/NatE), and TvArd1 (4pV6/NatA) show that the key catalytic residues that mediate recognition and acetylation of Met residue in Naa50p^31^ are conserved in both RimI^Mtb^ and TvArd1 (Supplementary Figure S5). V29 of Naa50p aligned completely with E34 of TvArd1 (Fig. 6a). However, according to LPC/CSU analysis, corresponding residue E25 of RimI^Mtb^ neither participated in substrate interactions nor aligned structurally with V29 and E34 of Naa50p and TvArd1, respectively. Rather, hydrogen bonding between N-terminal Met of substrate and Y140 of enzyme RimI^Mtb^ was found to be stabilizing (Fig. 6a). Residue Y140 of RimI^Mtb^ is conserved in Naa50p (Y139) and TvArd1 (Y141) and is previously known to be crucial for catalysis by Naa50p. Further, based on docking results and contact analysis, residues L28, F29, I82, L112 and A121 of RimI^Mtb^ provide a putative hydrophobic pocket for NatE like substrates (Fig. 6b). This pocket explains the higher specific activity of RimI^Mtb^ as observed towards substrates with a hydrophobic residue at N-termini (Fig. 4) and poor activity against substrates containing polar residues like Ser, Glu (Supplementary Figure S3). For efficient catalysis of NatA substrates, Glu35 is identified as an important residue in archaeal ortholog SsArd1. The same residue is attributed with lowering the acetylation efficiency of SsArd1 for Met N-termini due to steric hindrance^32^. Interestingly, this residue is conserved amongst prokaryotic NATs, TvArd1, SsArd1, and RimI^Mtb^ (Supplementary Figure S5, residues highlighted in red), yet RimI^Mtb^ is capable of efficient N-terminal Met acetylation of the substrate peptide.

Further, the shapes of the binding pocket of RimI^Mtb^, Naa50p and TvArd1 were analyzed by calculating surface area and volume of the binding site using CASTp server^33^. The calculated ratios of surface area to volume of the binding pockets of RimI^Mtb^, TvArd1 and Naa50p were 0.8, 0.83 and 0.57, respectively indicating deeper narrower sites for RimI^Mtb^ in comparison to that of Naa50p (Fig. 6c–e). The narrow tunnel shape of the substrate binding pocket suggests that the N-terminus of the substrate proteins needs to be accessible for Nα-acetylation, which could be the reason for the inability of RimI^Mtb^ to acetylate purified, folded proteins in our experiments.

### Curious genetic context of RimI^Mtb^ in the light of its protein-protein interactions

**Figure 7:**
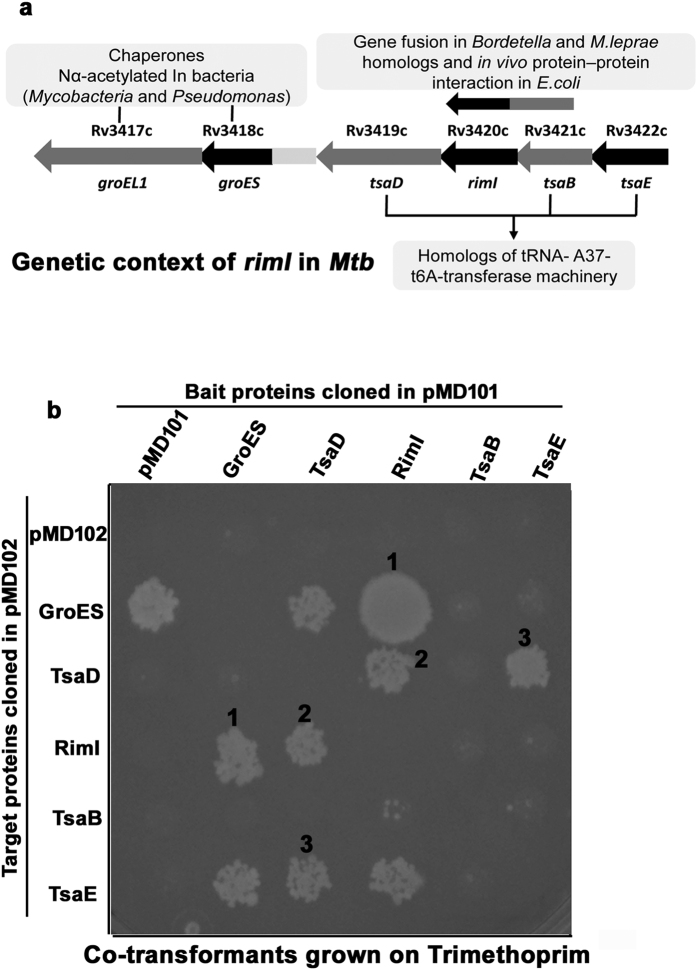
*In vivo* protein-protein interactions between RimI^Mtb^ and neighboring proteins. (**a**) Schematic representation of genetic context of *rimI* in *Mtb* (**b**) Mycobacterial Protein Fragment Complementation (MPFC) assay elucidating *in vivo* protein-protein interactions between 1) RimI^Mtb^ and GroES 2) RimI^Mtb^ and TsaD and 3) TsaD and TsaE, where corresponding co-transformants got selected on Trimethoprim supplemented at a concentration of 50 μg/ml.

As discussed under introduction section, *rimI* is positioned upstream of essential chaperone genes, *groES* and *groEL1* and packed in-between homologs of essential tRNA-A_37_-t^6^A transferase enzymes: *tsaD*, *tsaB*, *tsaE*. While chaperones are important for survival during various stress conditions, tRNA-A_37_-t^6^A modification of tRNA is universally conserved and considered critical for maintaining translational fidelity^23^. Though the context is quite conserved, the significance of *rimI* here is not understood yet. Interestingly, in eukaryotes, NatA is known to interact with a chaperone-like protein HYPK (Huntington Interacting Protein)^34^. HYPK assists co-translational Nα-acetylation activity of NatA and in its absence, Nα-acetylation decreases. Also, *rimI* is seen fused with *tsaB* in *Bordetella* and *Mycobacterium leprae* genomes and a weak *in vivo* interaction between RimI and TsaB is reported in *E. coli*, previously^35^ (Fig. 7a).

Therefore, employing a previously described Mycobacterial Protein Fragment Complementation (MPFC) assay^36^, we explored the *in vivo* interactions of RimI^Mtb^ with proteins of its conserved neighborhood. For the assay, *rimI,* two upstream genes: *tsaB, tsaE* and three downstream genes: *tsaD, groEL1 and groES* were cloned independently and separately in vectors namely, pMD101 and pMD102 that harbor complementary domains of murine dihydrofolate reductase (mDHFR) (Supplementary Tables S1 and S2). These recombinant vectors were then co-transformed in *Mycobacterium smegmatis* (*M. smegmatis*) in all combinations. A successful *in vivo* protein-protein interaction reconstitutes mDHFR in co-transformants and aids survival of cells on the otherwise inhibitory concentration of Trimethoprim. Accordingly, in our experiments, we observed reliable *in vivo* interactions between RimI^Mtb^ and GroES (the chaperone) as well as between RimI^Mtb^ and TsaD (the main actor protein of tRNA-A_37_-t^6^A transferase enzymes) (Fig. 7b). GroEL1 was observed to interact with all the neighboring proteins including the mDHFR of vector backbone (data not shown).The promiscuous interaction of GroEL1 with several proteins including mDHFR for its chaperone nature is well documented^37^.

Interestingly, we could not detect anticipated interaction between RimI^Mtb^ and TsaB and between TsaB and TsaD that is previously well characterized^35^, in our MPFC experiments. For the reason that we could successfully purify TsaD-TsaB as co-eluting protein complex from *E. coli*, it is possible that the fusion product of TsaB-DHFR adopts a conformation that is not accessible for protein-protein interaction in MPFC system. On the other hand, TsaD is not known to interact with TsaE directly but through the formation of a ternary complex in *E. coli* and *S. typhimurium*^38^,^39^. Again, we found this to be contrary to our system and observed a direct *in vivo* interaction between TsaD and TsaE. Similar to our observation, recently TsaD -TsaE interaction were observed *in vitro* in calorimetric experiments with purified proteins in *S. typhimurium* in the presence of ATP^38^. Nonetheless, our results indicate a plausible, biologically relevant involvement of RimI^Mtb^ with tRNA-A_37_-t^6^A transferase enzymes in mycobacteria and explain conservation of *rimI* genetic context, in part. Here observed cellular protein-protein interactions of RimI^Mtb^ may have far reaching implications or additional physiological relevance that needs to be explored further.

## Discussion

Protein Nα-acetylation is a co/post-translational modification that is implicated in a variety of cellular processes and regulation thereof, in eukaryotes, but it is little understood in bacteria. A host of Nα-acetylated proteins have been identified in the pathogen, *Mtb*^6^,^20^. Enzymes belonging to GNAT family of proteins that acetylate amino group of aminoglycosides are well known in mycobacteria. For example, Eis is a lysine acetyltransferase that acetylates DUSP16/MKP-7 in *Mtb* while its ortholog acetylates antibiotic kanamycin in *M. smegmatis*^40^. In the present work, we have characterized first protein/peptide Nα-acetyltransferase (RimI^Mtb^) of *Mtb*. In our study, we identify that RimI^Mtb^ is a monomer in solution. We have developed a quick tryptic peptide-based method (STPL) to identify novel substrate specificities of such enzymes. RimI^Mtb^ acetylates a wide variety of N-terminal residues including hitherto unknown residues like Leu, Tyr, Glu, and Asp, *in vitro*. However, significant enzyme activity is observed against hydrophobic N-termini only. Further, the N-terminus of peptides representing important/essential proteins of *Mtb* namely, chaperones: GroES and GroEL1 were acetylated by RimI^Mtb^. To conclude, *in vitro* enzyme assays showed that RimI^Mtb^ exhibits promiscuous acceptor substrate specificity which overrides the previous notion of specific or stringent substrate requirements of prokaryotic NATs. The only other known NAT of relaxed specificity is archaeal SsArd1 41. While acceptor specificity of SsArd1 is closer to eukaryotic NatA, RimI^Mtb^ acetylates substrate akin eukaryotic NatE substrates, more efficiently. These experimental observations were substantiated well by *in silico* modeling and peptide docking studies. In *Mtb* at least 12% of reported Nα-acetylome^6^ bears Nα-acetylated Met at N-terminus. Therefore, RimI^Mtb^ by its NatE like specificity could be the primary NAT responsible for acetylating such substrates in *Mtb*.

Additionally, we have attempted to investigate the significance of conserved genomic context of *rimI* using *in vivo* MPFC assays. Our results accordingly suggest hitherto unnoticed involvement of RimI^Mtb^ with essential chaperones and tRNA-A_37_-t^6^A transferase enzymes in *Mtb*. This study we believe is an important advance in the understanding of the process and proteins involved in Nα-acetylation in *Mtb*, in specific and bacteria, in general.

## Materials and Methods

### Chemicals and Reagents

All chemical reagents were purchased from Sigma-Aldrich unless otherwise noted. Materials used for protein production and purification were purchased from GE healthcare. Restriction enzymes, molecular ladders were purchased from NEB and Fermentas (Thermo Fisher Scientific). Synthetic peptides were purchased from GLS China.

### Cloning and expression of *Rv3420c*/*rimI* from *Mtb*

Gene encoding RimI homolog in *Mtb*, *Rv3420c* (accession no.: CCP46242.3) was PCR amplified from *Mtb* genomic DNA (BEI Resources) using primers 20FP and 20RP (Supplementary Table S1) and cloned in NcoI and HindIII restriction sites of pET28a (Novagen) and confirmed by sequencing. The C-terminal His-tagged construct, RimI^Mtb^pET28a was transformed in Lemo21 (DE3) (NEB # C2528H) cells for expression. The cells were grown at 37 °C till 0.6 O.D. was reached, induced with 0.1 mM Isopropyl-β-D-1thiogalactopyranoside (IPTG) and incubated overnight, in shaking condition at 200 rpm, at 16 °C. The cells were subsequently harvested and pellets stored at −80 °C.

### Purification of RimI^Mtb^ protein

The stored pellets were thawed and re-dissolved in Lysis buffer: (20 mM Tris pH 8.0, 1 M NaCl, 10% glycerol, 10 mM imidazole and 4 mM β –mercaptoethanol). The cells were sonicated and lysate so obtained centrifuged at 12000 rpm to remove debris. The supernatant was applied to Ni-NTA resin equilibrated with lysis buffer and protein was allowed to bind for one hour at 4 °C with gentle shaking. The beads were then washed with washing buffer (20 mM Tris pH 8.0, gradient of 1 M NaCl-300 mM NaCl, 10% glycerol and 30 mM imidazole). Bound protein was eluted in 10 ml of Elution buffer (20 mM Tris pH 8.0, 300 mM NaCl, 10% glycerol and 350 mM imidazole). A fraction of eluted protein was subjected to gel filtration chromatography using Superdex 75 10/300 GL column (GE healthcare, calibrated with GE LMW standards) with 20 mM Tris pH8.0, 300 mM NaCl, 10% glycerol and 4 mM β-mercaptoethanol. Fractions were collected, pooled and concentrated using Amicon 10 kDa molecular weight cut off filters. For enzyme assay, protein was dialyzed extensively at 4 °C in dialysis buffer (20 mM HEPES pH8.0, 150 mM NaCl, 10% glycerol, 2 mM DTT). The purified protein was cleaned up using ziptip C18 (Merck Millipore) to remove salts according to manufacturer’s protocol, and subjected to LC-ESI-MS (using Agilent 6550 iFunnel Q-TOF LC/MS) for intact mass analysis. RimI^Mtb^ was resolved using SDS-PAGE and in-gel trypsin digestion (Sigma # T6567) was carried out according to manufacturers’ protocol. The MS spectra so obtained were searched against *Mtb* protein database using online MASCOT server (http://www.matrixscience.com/).

### Qualitative acetyltransferase activity assay (NAT assay)

For determining acetyltransferase activity of the enzyme, peptides consisting of first six amino acid residues (without the initiator Met) of putative protein substrates were synthesized (listed in Table 1). 100 μM of each peptide was added to a reaction mixture consisting of 20 mM HEPES pH 8.0, 100 μM acetyl coA and 4 μM RimI^Mtb^ protein. Reactions controls for the assays included, a) Substrate control A (Peptide alone), b) Substrate control B (Peptide + acetyl coA) and c) Enzyme control (Peptide + acetyl coA + dummy protein BSA). The reactions were incubated at 25 °C in a water bath, overnight. The samples were cleaned up using ziptip C18 and subjected to MALDI-TOF MS analysis (using AB SCIEX TOF/TOF™ 5800). The data was acquired using following settings: 1 μL of analyte was combined with 1 μL of the matrix (α-cyano-4-hydroxy-cinnamic acid matrix in 50% ACN/50% water with 0.1% TFA) and spotted onto an MALDI target and dried under ambient conditions before analysis. MS data was acquired in positive ion mode using fixed laser intensity of 3400, keeping the mass range 600–4000 Da, the total number of shots 2000, bin size 0.5 ns and pulse rate 400 Hz. MS/MS data was acquired using fixed laser intensity of 4300 keeping the mass range 400–700 Da and adduct tolerance 0.03. Precursors having S/N below 20 and resolution within 200 were excluded. The data obtained (t2d files) was analyzed by Data Explorer software (version 4.6) and mass peaks plotted in Origin (OriginPro 2015 b.9.2.214).

### Quantitative acetyltransferase assay (DTNB based)

DTNB assay or Ellman’s assay is a colorimetric assay that detects generation of free sulfhydryl group. In an acetyltransferase reaction, the products formed are acetylated substrate and CoASH. This free CoASH reacts with DTNB to form mixed disulfide and TNB which gives a yellow color at 412 nm. Thus, enzyme assays were set up according to published protocol^42^,^43^ along with control reactions a) Substrate control (Peptide + acetyl coA) b) Enzyme control (acetyl coA + RimI^Mtb^). Briefly, reaction components were added in 50 mM Tris Buffer pH 8.0, 4 mM peptide substrate, 0.5 mM acetyl coA in a total reaction volume of 0.1 ml. Reactions were initiated by addition of 8 μM of RimI^Mtb^ and samples incubated at 25 °C for one hour. Enzyme activity was quenched by addition of 60 μl of 3.2 M Gn-Hcl and 10 μl of 2 mM DTNB was added. The reactions were vortexed and allowed to stand for 5 min before absorbance was measured at 412 nm in a plate reader. Absorbance of enzyme control was subtracted from enzyme assay readouts. For each peptide, the amount of product formed was calculated from CoASH calibration curve (0.1 mM–1 mM) and specific activity of the enzyme calculated. The mean average value and standard deviation for triplicate experiments were plotted.

### Donor specificity assay

For determining donor substrate specificity, RimI^Mtb^ was incubated with 100 μM of peptide substrate (DP8) and 500 μM each of acetyl coA, succinyl coA and propionyl coA at 25 °C overnight. Data was acquired as mentioned above.

### Small-scale Tryptic Peptide Library assay (STPL assay)

#### Trypsin digestion

10 μg of each purified, mass-verified protein was reconstituted in 100 μl of 6 M urea and trypsin digested according to manufacturers’ protocol. Briefly, the samples were reduced by addition of DTT and kept at 37 °C for one hour. The samples were cooled down and alkylated using Iodoacetamide and again kept at incubation for one hour at RT in dark. 20 mM of ammonium bicarbonate (pH 8.0) was added to dilute concentration of urea to 1 M. Trypsin (Sigma: T6567) was added in a ratio of 1:50 and incubated at 37 °C overnight. The samples were speed vacuumed to 100 μl and ziptip purified.

#### Enzyme assay (NAT assay)

For each set of proteins, one control reaction consisting of peptides generated by digest and acetyl coA and another enzyme reaction consisting of the tryptic digest, acetyl coA and RimI^Mtb^ protein was set up in 20 mM HEPES buffer pH 7.5. The reactions were incubated at 25 °C overnight, ziptip purified and subjected to PMF analysis by MALDI.

#### Database search

Each control and enzyme MS spectra obtained was searched using online MASCOT Server against Mycobacterium complex in Swiss-Prot database with single missed cleavage of trypsin allowed. Carbamidomethylation of Cys (+57) was set as fixed modification while Nα-acetylation (+42) was set as variable modification. Mass tolerance for precursor ion was set as 0.2 Da and MASCOT report was set at AUTO to obtain only the protein/peptide hits with significant scores (threshold score for identified peptide was >30).

### *In vivo* protein-protein interaction studies: MPFC

*rimI* and its neighboring genes namely, *Rv3417c, Rv3418c, Rv3419c, Rv3421c, Rv3422c* were PCR amplified from *Mtb* genomic DNA using respective primers (Supplementary Table S1) and cloned in EcoRI –HindIII and BamHI- HindIII sites of pMD101 (Kan^R^) and pMD102 (Hyg^R^), respectively. The constructs (Supplementary Table S2) were co-transformed in all possible combinations in *M. smegmatis* mc^2^155 electrocompetent cells and grown on LB medium containing Kanamycin and Hygromycin (LB-Kan + Hyg) at concentration 50 μg/ml. The co-transformants were selected on LB-Kan + Hyg plates containing Trimethoprim (Trim) at conc. of 50 μg/ml to decipher positive interaction between corresponding proteins. Co-transformants bearing (pMD101 + pMD102), (pMD101 + pMD102-test gene fusion) and (pMD102- test gene fusion + pMD101) served as negative controls.

### Statistical analysis

All statistical analysis was performed using one-way ANOVA with Dunnett’s multiple comparison test.

## Additional Information

**How to cite this article**: Pathak, D. *et al*. Biochemical evidence for relaxed substrate specificity of Nα-acetyltransferase (*Rv3420c*/*rimI*) of *Mycobacterium tuberculosis*. *Sci. Rep.* **6**, 28892; doi: 10.1038/srep28892 (2016).

## Supplementary Material

Supplementary Information

## Figures and Tables

**Table 1:** List of acceptor peptides used as substrates for *in vitro* NAT assays.

Substrate Protein	Gene Name (Organism)	Abbreviation	Peptide Sequence	Specificity
Ribosomal proteins	*rpsRS18* (*S. typhimurium*)	DPC	ARYFRR	NatA like
	*rpsR1*/*Rv0055* (*Mtb*)	DP1	AKSSKR	–
	*rpsR2*/*Rv2055* (*Mtb*)	DP2	AAKSAR	–
Neighboring proteins	*tsaB*/Rv3421c (*Mtb*)	DP4	SRVQIS	NatA like
	*tsaE*/*Rv3422c* (*Mtb*)	DP5	SREGIR	–
	*groEL1*/*Rv3417c* (*Mtb*)	DP6	SKLIEY	NatA like
	*groES*/*Rv3418c* (*Mtb*)	DP7	AKVNIK	NatA like
Custom synthesized peptides		DP8	LRYFRR	Novel
		DP9	MARYFRR	NatE like
		DP10	MERYFRR	NatB like
		DP11	MLRYFRR	NatC like
