# Supplementary material for: Biochemical evidence for relaxed substrate specificity of Nα-acetyltransferase (Rv3420c/rimI) of Mycobacterium tuberculosis
Source: Sci Rep. 2016 Jun 29;6:28892. doi: 10.1038/srep28892 (PMC4926160; doi:10.1038/srep28892)
Supplement: Supplementary Information [file srep28892-s1.pdf]

**Supplementary Information**

# Biochemical evidence for relaxed substrate specificity of Nα-acetyltransferase (*Rv3420c*/ *rimI*) of *Mycobacterium tuberculosis*

**Deepika Pathak[1, 3], Aadil Hussain Bhat[1, 3], Vandana Sapehia[1], Jagdish Rai[2] and Alka Rao[1*]**

[1] CSIR-Institute of Microbial Technology, Sector 39-A, Chandigarh-160036, India

[2] Institute of Forensic Science & Criminology, Panjab University, Sector 14, Chandigarh-160014, India

[3]Co-first authors

[*]Correspondence to Alka Rao, CSIR-Institute of Microbial Technology, Sector 39-A, Chandigarh-160036, India. Email: raoalka@imtech.res.in

a

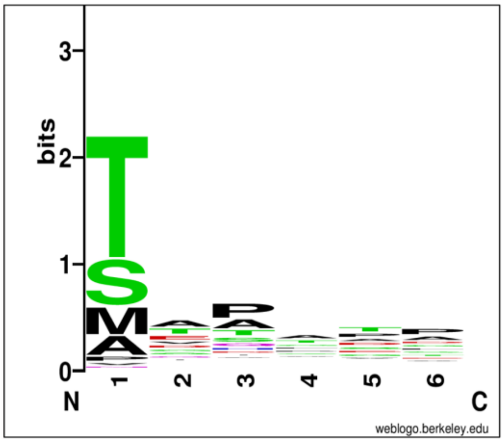


b

| Specificity | Abundance |
|---|---|
| **NatA** (-Ala,-Ser,-Gly,-Cys,-Thr,-Val,-Asp,-Glu) | 82.40% |
| **NatB** (Met-Asn, Met-Asp, Met-Gln, Met-Glu) | 2.50% |
| **NatC** (Met-Ile,Met-Leu, Met-Phe,Met-Trp) | 1.60% |
| **NatD** (Specific to Histones) | - |
| **NatE** (-Met) | 7.11% |
| **Unique N-termini** (-Asn,-Ile, -Phe) | 4.6% |

**Figure S1. Sequence analysis of Nα-acetylome of *Mtb*[1].**(a) Sequences of Nα-acetylated proteins were analysed using Web logo server[2].The web logo indicates the abundance of various acetylated N-termini in *Mtb* acetylome, where the order of abundance is Thr (54%), Ser (19%) and Met (12%) (b) Abundance of different eukaryotic NAT–type substrates in *Mtb* proteome as deciphered from acetylated N-termini sequences.

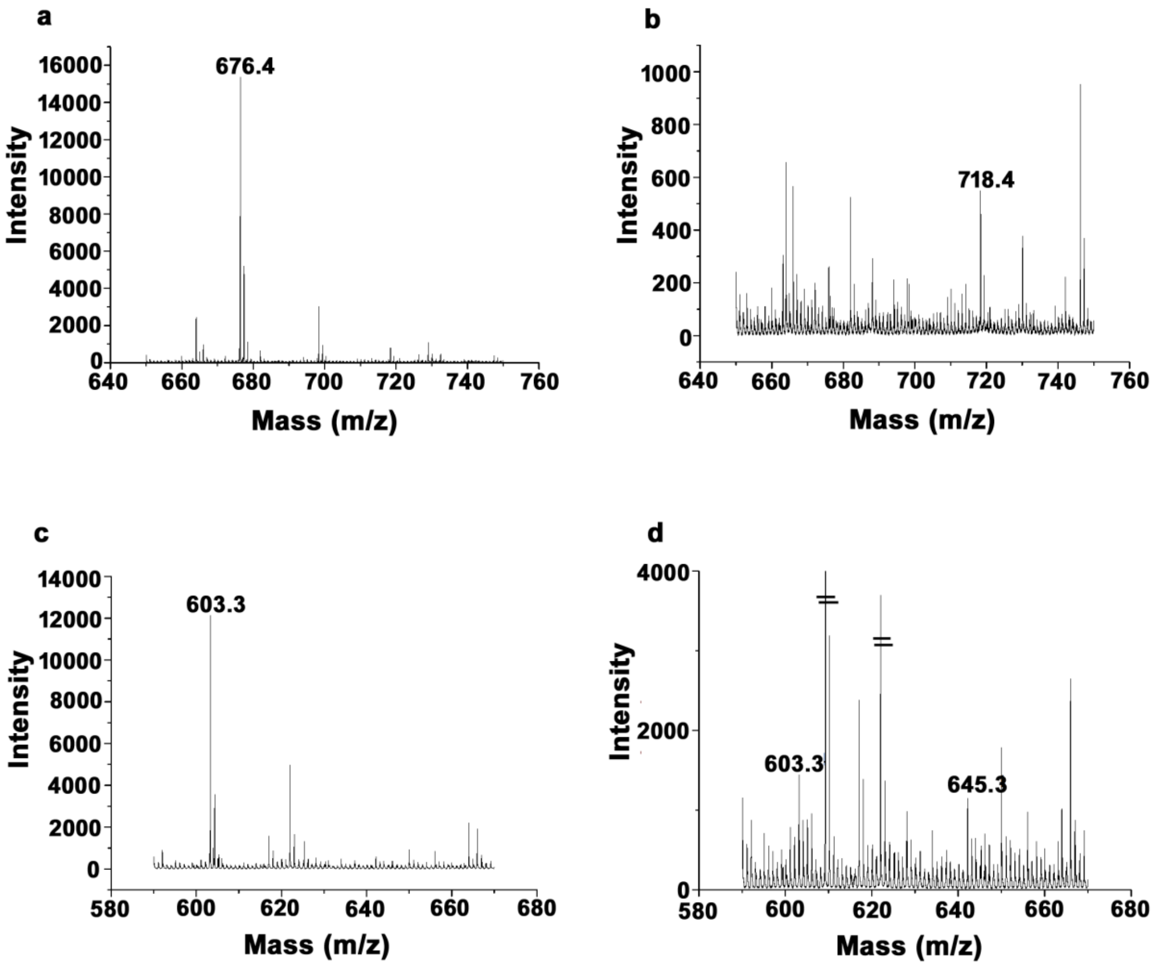


**Figure S2. NAT assay of *Mtb* ribosomal proteins rpsR1 (peptide DP1) and rpsR2 (peptide DP2).** MALDI-MS of control reaction (a) showing unmodified substrate DP1 (676.4 Da, S/N = 1729) and enzyme reaction (b) where substrate DP1 (718.4 Da, S/N=90) is poorly modified in the presence of enzyme RimI$^{Mtb}$. Similarly, MALDI-MS of control reaction (c) enzyme reaction (d) showing unmodified substrate DP2 (603.4 Da, S/N ratio =837) and poorly modified substrate DP2 (645.4 Da, S/N ratio=22), respectively in the presence of enzyme RimI$^{Mtb}$.

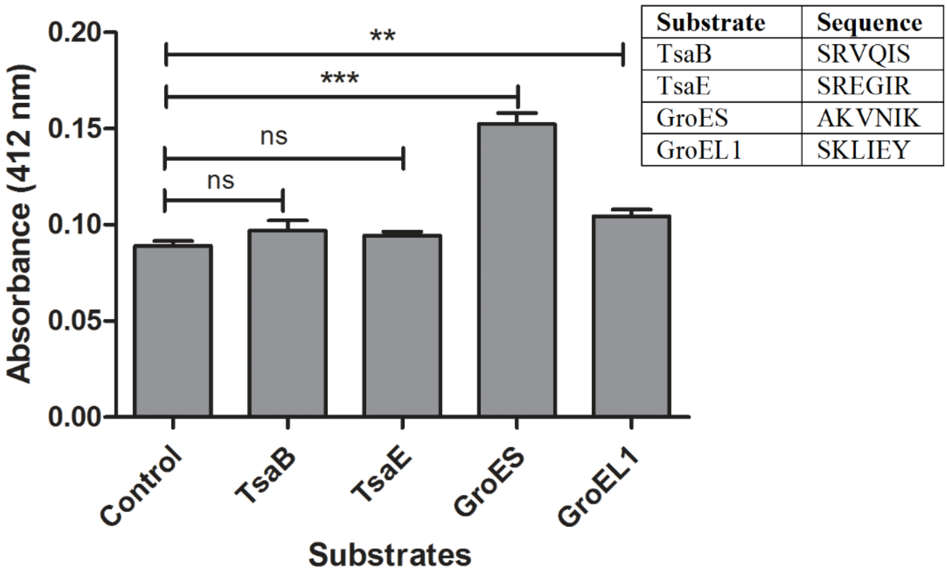


| Substrate | Sequence |
|---|---|
| TsaB | SRVQIS |
| TsaE | SREGIR |
| GroES | AKVNIK |
| GroEL1 | SKLIEY |

**Figure S3. Quantitation of acetylated products generated by RimI$^{Mtb}$ using DTNB assay[3].** The amount of product formed was quantified by measuring the absorbance of each reaction at 412 nm. Significant specific activity was observed against GroES peptide alone (231.69 µmoles/mg/min as calculated from DTNB standard curve. Control reaction consisted of RimI$^{Mtb}$ and acetyl coA without acceptor peptide. The results shown here represent mean ± SD of experiments performed in triplicate (n=3). Statistical significance of each result was obtained using one-way ANOVA with Dunnett's multiple comparison test. The significance is represented by asterisks, ** for p value < 0.01, *** for p value < 0.001. P value < 0.05 was considered significant; ns for not significant.

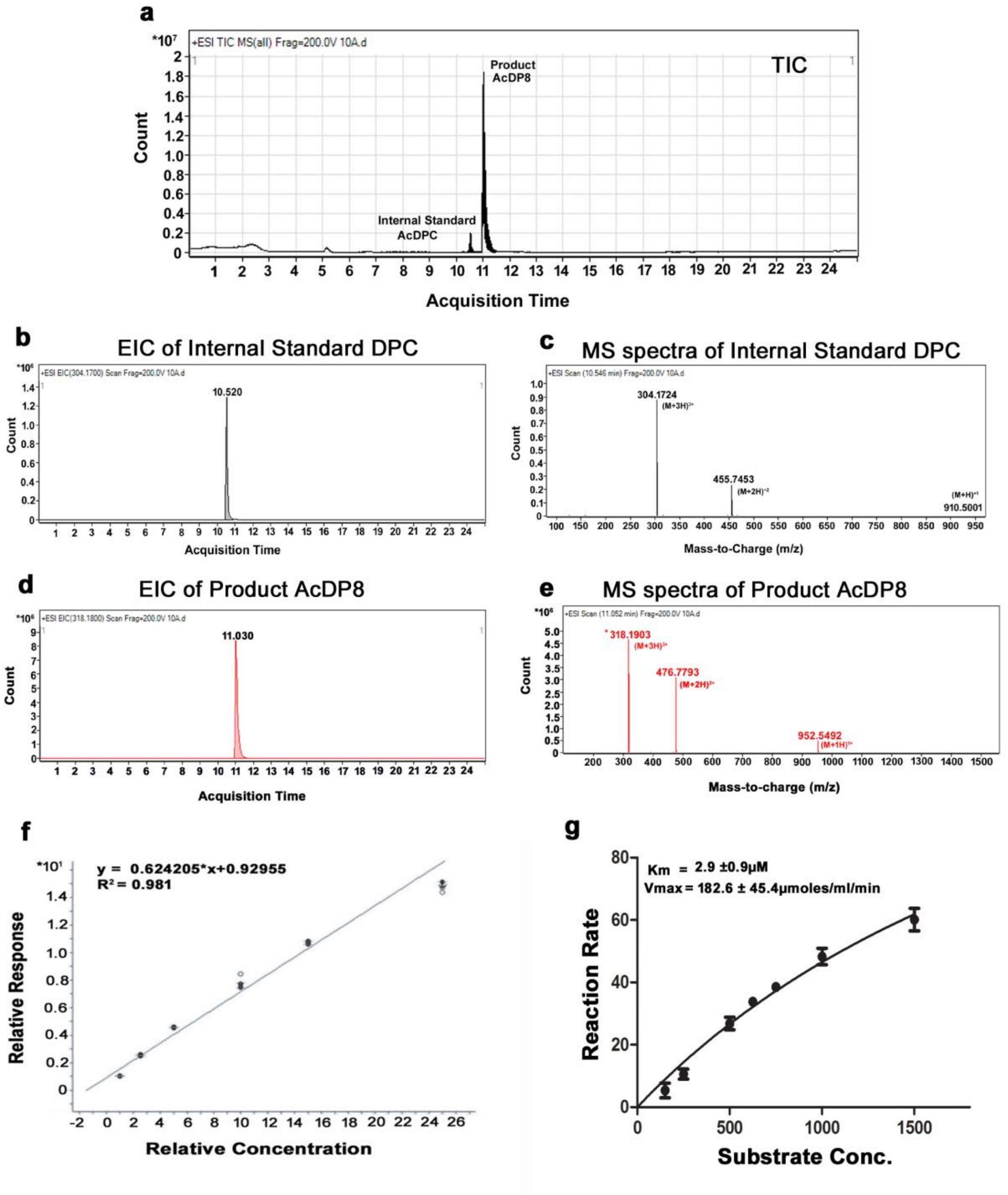


**Figure S4. Selected ion monitoring (SIM) to determine kinetic parameters of RimI$^{Mtb}$.** (a) Total ion count (TIC) of custom synthesized Nα-acetylated DPC (Ac-DPC, internal standard) and Nα- acetylated DP8 (Ac-DP8, Product). (b) Extracted ion chromatogram (EIC) of internal standard (Ac-DPC), showing elution of internal standard at 10.5 min. (c) MS spectra of internal standard showing triply charged precursor ion of internal standard having m/z of 304.17Da as most abundant ion (used for internal standard quantitation). (d) EIC of product (Ac-DP8): Ac-DP8 that eluted at 11.03 min. (e) MS spectra of product showing triply charged precursor ion of m/z 318.19Da as most abundant ion (used for product quantitation). (f) Calibration curve plotted by MSQuant software using Ac-DP8 as standard and Ac-DPC as internal standard. (g) Michaelis-Menten kinetic plot for RimI$^{Mtb}$. The values in (f) and (g) represent mean ± SD of two experiments (n=2).

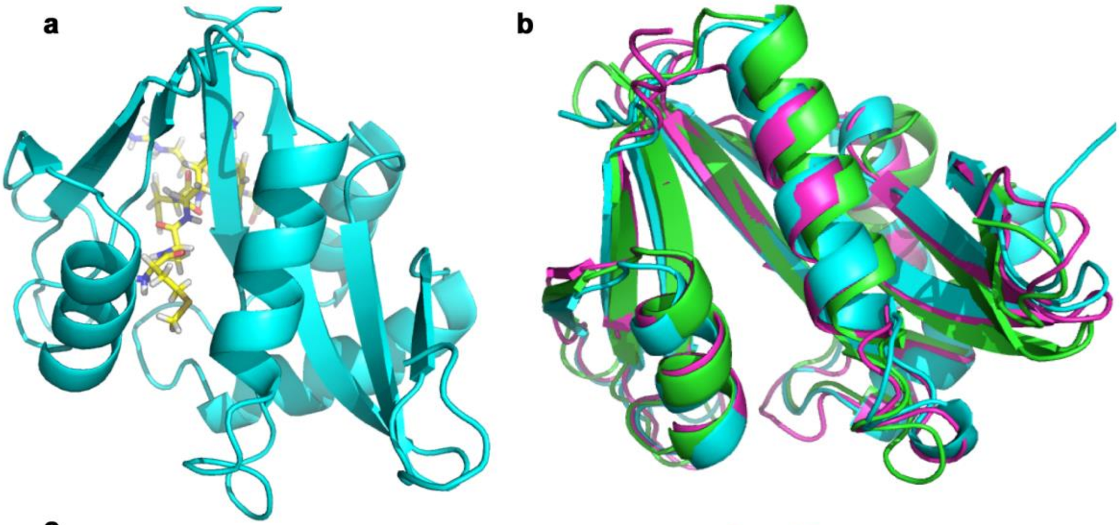


c

```
RimI          ---MEVTA----------DTEPVTIGALTRADAQRCAELEAQLFVG--DDPWPPAAFNRE
Naa50         ---------MKGSRIELGDVTPHNIKQLKRLNQVIFPVSYNDKFYKDVLEVGELAKLAYF
TvArd1        MVYMAIN----AVAGTIREFSPKDIESVYRIAQTSLTEYYTQALILDLHREWPESFMVYT
Ssard1        ---MELAEKDKGRDFTLRNARMDDIDQIIKINRLTLPENYPYYFFVEHLKEYGLAFFVAI
                                :     *  : :             :          : :

RimI          LASPHNHYVGARSGGTLVGYAGISRLGRTPPFEYEVHTIGVDPAYQGRGIGRRLLRELLD
Naa50         ----NDIAVGAVCCRVDHS--------Q-NQKRLYIMTLGCLAPYRRLGIGTKMLNHVLN
TvArd1        V---AGSVVGFIVGSKYS------------RTEARILLFAVDERFRRMGVGSALMDAFLS
Ssard1        V---DNSVVGYIMPRIEWGFSNIKQLPS-LVRKGHVVSIAVLEEYRRKGIATTLLEASMK
                      **                      .  :  :.    ::  *:.  ::   :.

RimI          FAR----GGVVYLEVRTDNDAALALYRSVGFQRVGLRRRYYRVSGADAYTM--RRDSGDP
Naa50         ICEKDGTFDNIYLHVQISNESAIDFYRKFGFEIIETKKNYYKR-IEPADAHVLQKNLKVP
TvArd1        -LCREQNMLSVRLEVRTDNDEAIRFYKKYGFVITAMLPNYYSD-SSNAYTM--WRIVLEH
Ssard1        SMKNDYNAEEIYLEVRVSNYPAIALYEKLNFKKVKVLKGYYAD-GEDAYLM--ARPL---
                        : *.*: .*  *: :*..  *        **      *      :

RimI          SKLAAALEHHHHHH
Naa50         SGQNADVQKTDN--
TvArd1        HHHHH---------
Ssard1        --------------
```

d

| Key catalytic residues conserved between Naa50p, TvArd1 and RimI$^{Mtb}$ | | |
|---|---|---|
| **Naa50p** | **TvArd1** | **RimI$^{Mtb}$** |
| V29 | E34 | E25 |
| H112 | E114 | E113 |
| Y138 | Y140 | Y139 |
| Y139 | Y141 | Y140 |

**Figure S5. Comparative analysis of structures and sequences of Naa50p, TvArd1 and RimI$^{Mtb}$.** (a) RimI$^{Mtb}$ model docked with peptide DP9 (MARYFRR) (b) RimI$^{Mtb}$ model (cyan) superimposed with crystal structures of Naa50p (green), TvArD1 (magenta) (c) Sequence comparison between Naa50p, RimI$^{Mtb}$, TvArd1 and SsArd1 using Clustal Omega. The residues highlighted in red are catalytic residues, green are hydrophobic residues and yellow highlights ‘P-loop’ -acetyl-coA binding region (d) Key residues that are conserved between three NATs based on sequence and structural alignment.

## Table S1. Primers used in this study

| Gene | Primers | Common Name |
|---|---|---|
| **Rv3420c** | FP:5’TACGTAACCATGGAAGTGACGGCCGACACCGAGCC 3’<br>RP: 5’ TACGTAAAGCTTTGACGGGTCCCCCGAATCCC 3’ | 20FP<br>20RP |
| **Rv3418c** | FP: 5’ TACGTAACATATGGTGGCGAAGGTGAACATC 3’<br>RP: 5’TACGTAAAAGCTT CTT GGAAACGACGGCCAGCA 3’ | 18FP<br>18RP |
| **Rv3421c** | FP: 5’TAGGTGGCATATGAGCCGCGTGCAAATAAG<br>RP: 5’TACGTAAAGCTTCGTGCAAACCGCCAGCGGCT | 21FP<br>21RP |
| **Rv3421-<br>Rv3419c** | FP:5’ TACGTAAGGATCCAATGAGCCGCGTGCAAATAAG 3’<br>RP:5’ TACGTAAAGCTTTCACGTGCAAACCGCCAG 3’<br>FP: 5’ TACGTAACATATGACGACAGTCTTGGGCATCG3’<br>RP: 5’TACGTACTCGAGTCACCGCACCTGCCCCTGCA 3’ | 21DTBF<br>21DTBR<br>19DTF<br>19DTR |
| **Rv3417c** | FP:5’ TACGTAAGAATTCA AGCAAGCTGATCGAATA 3’<br>RP: 5’TACGTAAATCGATTCAGTGCGCGTGCCCGTG 3’ | 17pMD101F<br>17pMD102R |
| **Rv3418c** | FP: 5’ TACGTAAAAGCTT AGTGGCGAAGGTGAACATC 3’<br>RP:5’ TACGTAAATCGATTCACTACTTGGAAACGACGGC 3’ | 18pM102F<br>18pMD102R |
| **Rv3419c** | FP:5’TACGTAAAAGCTTAATGACGACAGTCTTGGGCATCG 3’<br>RP: 5’TACGTAAATCGATTCACCGCACCTGCCCCTGCA 3’ | 19pMD102F<br>19pMD102R |
| **Rv3420c** | FP: 5’TACGTAAGAATTCAGTGACGGCCGACACCGAG 3’<br>RP:5’ TACGTAAAGCTTTCATGACGGGTCCCCCGAAT 3’ | 20pMD101F<br>20DTR |
| **Rv3420c** | FP: 5’ TACGTAAGGATCCGTGACGGCCGACACCGAG 3’<br>RP:5’ TACGTAAAGCTTTCATGACGGGTCCCCCGAAT 3’ | 20pMD102<br>20DTR |
| **Rv3421c** | FP: 5’TACGTAAGAATTCAATGAGCCGCGTGCAAATAAG3’<br>RP: 5’ TACGTACTCGAGTCACGTGCAAACCGCCAG 3’ | 21pMD101F<br>21DTBR |
| **Rv3421c** | FP: 5’ TACGTAAGGATCCATGAGCCGCGTGCAAATAAG 3’<br>RP: 5’ TACGTACTCGAGTCACGTGCAAACCGCCAG 3’ | 21pMD102<br>21DTBR |
| **Rv3422c** | FP: 5’ TACGTAAGAATTCATTGAGCCGTGAGGGTATCCG 3’<br>RP: 5’ TACGTAAAGCTTTCATGACCGGCCCCACGACCA 3’ | 22pMD101F<br>22DTR |
| **Rv3422c** | FP: 5’ TACGTAAGGATCCTTGAGCCGTGAGGGTATCCG 3’<br>RP: 5’ TACGTAAAGCTTTCATGACCGGCCCCACGACCA 3’ | 22pMD102<br>22DTR |

## Table S2. Strains and Plasmids used in this study

| **Bacterial strain or plasmid** | **Description** | **Source or reference** |
|---|---|---|
| *Escherichia coli* TOP10 | F$^-$ *mcrA* Δ(*mrr-hsdRMS-mcrBC*) ϕ80*lacZ*ΔM15 Δ*lacX74 recA1 araD139* Δ(*ara leu*)*7697 galU galK rpsL* (*StrR*) *endA1 nupG* | Lab Collection |
| *Escherichia coli* Lemo BL21(DE3) | *Δ(mcrA) 183 Δ(mcrCB-hsdSMR-mrr) 173 endA1 supE44 thi-1 recA1 gyrA96 relA1 lac* | Stratagene |
| *Mycobacterium smegmatis* mc$^2$155 | High-transformation mutant of *M. smegmatis* ATCC 607 | ATCC 700084 |
| RimI$^{Mtb}$pET28a | 489 bp of Rv3420c (RimI$^{Mtb}$) amplified using primers 20F and 20R and cloned in NcoI and HindIII restriction sites of expression vector pET28a | This study |
| Rv3418pNIC28a | 304 bp of Rv3418c amplified using primers 18FP and 18RP and cloned in NdeI and HindIII restriction sites of expression vector pNIC28a | This study |
| Rv3421pNIC28a | 636 bp of Rv3421c amplified using primers 21FP and 21RP and cloned in NdeI and HindIII restriction sites of expression vector pNIC28a. | This study |

| | | |
|---|---|---|
| Rv3421-19pETDuet-1 | 636 bp of Rv3421c amplified using primers 21DTBF and 21DTBR and cloned in NcoI and HindIII restriction sites of expression vector pETDUET-1. 1035bp of Rv3419c amplified using primers 19DTF and 19DTR and cloned in NdeI and XhoI restriction sites of Rv3421pETDuet-1. | This study |
| pUAB400/pMD101 plasmid | Integrative mycobacteria – *E. coli* shuttle plasmid, Kan$^r$ | [4] |
| pUAB300/pMD102 Plasmid | Episomal mycobacteria – *E. coli* shuttle plasmid, Hyg$^r$ | [4] |
| Rv3417pMD101 | 1627bp cloned in pMD101 vector using primers 17pMD101F and 17pMD102R | This study |
| Rv3418pMD101 | 304bp cloned in pMD101 vector using primers 18pMD101F and 18pMD102R | This study |
| Rv3419pMD101 | 1035bp cloned in pMD101 vector using primers 19pMD101F and 19pMD102R | This study |
| Rv3420pMD101 | 489bp cloned in pMD101 vector using primers 20pMD101F and 20DTR | This study |
| Rv3421pMD101 | 636bp cloned in pMD101 vector using primers 21pMD101F and 21DTBR | This study |
| Rv3422pMD101 | 507bp cloned in pMD101 vector using primers 22pMD101F and 22DTR | This study |
| Rv3417pMD102 | 1627bp cloned in pMD102 vector using primers 17pMD101F and 17pMD102R | This study |
| Rv3418pMD102 | 304bp cloned in pMD102 vector using primers 18pMD101F and 18pMD102R | This study |
| Rv3419pMD102 | 1035bp cloned in pMD102 vector using primers 19pMD101F and 19pMD102R | This study |
| Rv3420pMD102 | 489bp cloned in pMD102 vector using primers 20pMD102F and 20DTR | This study |
| Rv3421pMD102 | 636bp cloned in pMD102 vector using primers 21pMD102F and 21DTBR | This study |
| Rv3422pMD102 | 507bp cloned in pMD102 vector using primers 22pMD102F and 22DTR | This study |

**Table S3. A quantitative comparison of ratios of MS intensities of Product: Substrate (P: S) ions of custom substrates.** All the NAT assays were carried out and processed under identical conditions. Theoretical ionization efficiencies are similar by virtue of sequence similarity. The ratios (P: S) were derived from absolute MS intensities obtained from MALDI-MS.

| **Substrate name** | **Peptide Sequence** | **Enzyme reaction (P: S)** | **Control reaction (P: S)** |
|---|---|---|---|
| DPC (NatA substrate) | ARYFRR | 0.97 | 0.03 |
| DP8 (Novel substrate) | LRYFRR | 1.07 | 0.131 |
| DP9 (NatE substrate) | MARYFRR | 18 | 0.027 |
| DP10 (NatB substrate) | MERYFRR | 0.240 | 0.079 |
| DP11 (NatC substrate) | MLRYFRR | 0.866 | 0.033 |

## Supplemental Methods

Selected ion monitoring to determine kinetic parameters of $RimI^{Mtb}$

Chromatographic conditions:

Separation of peptides was achieved on Xbridge BEH C18 column (200mm, 4.6mm, 5µm, and 200A), using UHPLC Agilent Technologies 1290 infinity system with TCC (Thermal column compartment). 1.5µl of each sample was injected through an autosampler. A gradient program from 2% to 90% eluent B over 25min run at a flow rate of 300µl/min was used to separate unmodified and modified peptides.

Mass spectrometry

All mass spectra were acquired on Agilent 6550 i-funnel Q-TOF LC/MS equipped with an Agilent Dual jet-stream ESI source. MassHunter workstation software version (B.05.00) by Agilent Technologies was used for data acquisition. During the sample analysis, the source parameters were operated using the following settings, drying gas: 13 L/min (220$^{o}$ C), sheath gas: 11 L/min (320$^{o}$ C), VCap: 4000 V, skimmer: 65V, fragmentor: 200 V, nebulizer: 35 psig. Peptides were fragmented using ramped collision energy. MS spectra were acquired in the range of 100 m/z to 2000 m/z and MS and MS/MS scan rate of 2 spectra/sec and 4 spectra/sec, respectively. The fragmentation of precursor ions were performed by using Targeted MS/MS.

Enzyme assay

The kinetic parameters of $RimI^{Mtb}$ were measured by monitoring the product, i.e, acetylated DP8 peptide (LRYFRR). Acetylated–DPC (Ac-ARYFRR) was chosen as the internal standard because of similar ionization efficiency to the product. Reaction mixtures consisted of 20mM ammonium bicarbonate buffer (pH 7.5), acetyl-coA, DP8 peptide (substrate), Ac-DPC (IS) and $RimI^{Mtb}$. HPLC method was optimized for separation of substrate and product and Linearity of the progress curve was determined at different concentration of enzyme and at different time points. To determine the Km and Vmax for peptide, varying concentrations of peptide were chosen (50µM-1500µM) at a fixed saturating concentration of acetyl-coA (500µM) with a constant concentration (10µM) of the internal standard. Enzyme reactions were initiated by addition of 1µM of enzyme in a 50µl reaction and kept at 25°C for 1hour. The reaction was stopped by addition of 0.1% formic acid. The quenched reaction mixtures were subsequently analysed by LC/MS.

Quantitation and Data Analysis

MS analysis was performed in positive ion mode. To determine the order of elution of peptides in reaction mixtures, Ac-DP8 and Ac-DPC (IS) were mixed and analysed in full scan mode. The retention time for Ac-DP8 was found to be 10.96 min and that of Ac-DPC was found to be 10.46 min.  Triply charged precursor ions for both product and internal standard, having m/z 318.18 and 304.17 respectively, were found to be predominant in the spectra and consequently selected for SIM analysis as qualifier ions. Their product ions having m/z 86.1 and 70.1 respectively were set as quantifier ions. For selected ion monitoring (SIM), the intensity ratio of product and the internal standard was monitored during standard preparation and quantification of product concentration in sample. Data was acquired in targeted MS/MS mode and a seven - point calibration curve of product Ac-DP8 plotted using the MSQuant software (B.05.00) and subsequently utilized for calculating concentration of product in reaction mixtures. Calibration curve was generated from $Ip/I_{IS}$ versus [product]/[IS] plots. Slope of the line m was calculated such that [P] conc. can be determined using equation: $[\text{Product}] = (IP / I_{IS}) * [\text{IS}] / m$. Velocity of the reaction was calculated and plotted against substrate concentration using Graphpad Prism software and Miachelis –Menten parameters determined.

## References


1. Kelkar, D. S. *et al.* Proteogenomic Analysis of Mycobacterium tuberculosis By High Resolution Mass Spectrometry. *Mol. Cell. Proteomics* **10,** M111.011627–M111.011627 (2011).

2. Crooks, G. E., Hon, G., Chandonia, J.-M. & Brenner, S. E. WebLogo: a sequence logo generator. *Genome Res.* **14,** 1188–90 (2004).

3. Angelis, J., Gastel, J., Klein, D. & Cole, P. ENZYMOLOGY : Kinetic Analysis of the Catalytic Mechanism of Serotonin N- Kinetic Analysis of the Catalytic Mechanism of Serotonin. *Na* **273,** 3045–3050 (1998).

4. Singh, A., Mai, D., Kumar, A. & Steyn, A. J. C. Dissecting virulence pathways of Mycobacterium tuberculosis through protein-protein association. *Proc. Natl. Acad. Sci. U. S. A.* **103,** 11346–11351 (2006).
